# Supplementary material for: Spatial proteomics defines the content of trafficking vesicles captured by golgin tethers
Source: Nat Commun. 2020 Nov 25;11:5987. doi: 10.1038/s41467-020-19840-4 (PMC7689464; doi:10.1038/s41467-020-19840-4)
Supplement: Supplementary file 1 — Supplementary Information [file 41467_2020_19840_MOESM1_ESM.pdf]

Supplementary Information

Spatial proteomics defines the content of trafficking vesicles captured by golgin tethers

John J.H. Shin[1]*, Oliver M. Crook[2,3,4], Alicia C. Borgeaud[1], Jérôme Cattin-Ortolá[1], Sew-Yeu Peak-Chew[1], Lisa M. Breckels[3], Alison K. Gillingham[1], Jessica Chadwick[1], Kathryn S. Lilley[2,3], Sean Munro[1]*

[1]MRC Laboratory of Molecular Biology, Francis Crick Avenue, Cambridge, CB2 0QH, UK

[2]The Milner Therapeutics Institute, University of Cambridge, Puddicombe Way, Cambridge, CB2 0AW, UK

[3]Cambridge Centre for Proteomics, Dept. of Biochemistry, University of Cambridge, Cambridge, CB2 1QR, UK

[4]MRC Biostatistics Unit, Cambridge Institute for Public Health, Cambridge, CB2 0SR, UK

*Correspondence. Email: shin.jaehee@gmail.com (J.J.H.S.); sean@mrc-lmb.cam.ac.uk (S.M.)

Contents:

Supplementary Figures 1-4

Supplementary Tables 1 and 2

Supplementary Data 1 and 2 are provided separately as .xlsx files

Supplementary Movie 1 is provided separately as a .mov file

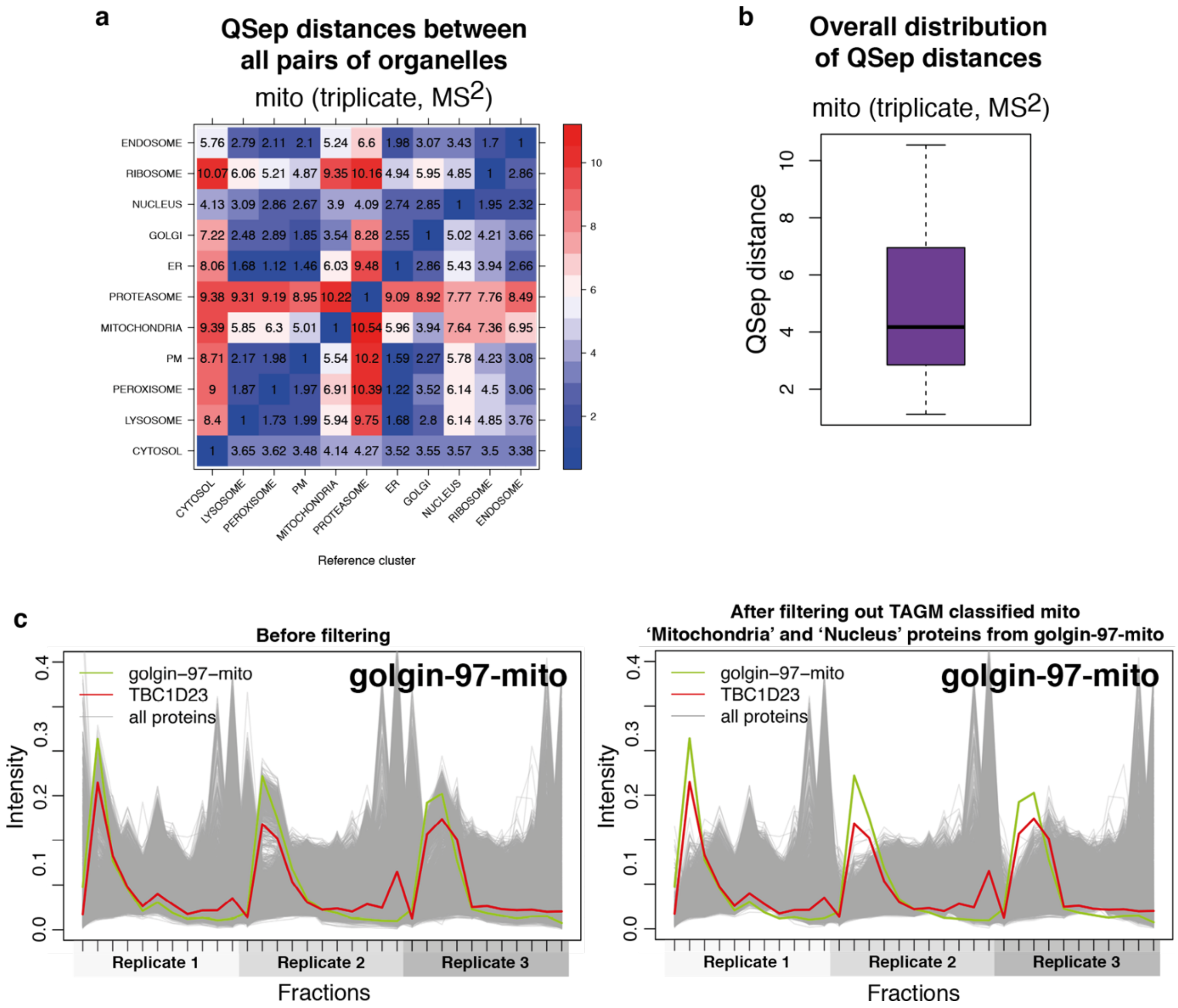

Supplementary Figure 1. Assessing and pre-filtering LOPIT-DC data.

a. QSep distance quantifying separation between all pairs of compartments in the LOPIT-DC of the mito control cell line. **b.** Boxplot of the overall distributions of QSep distances in (a), showing minimum, maximum, median and quartiles. **C.** TMT reporter ion distributions of proteins across fractions for each replicate for golgin-97-mito showing the effect of discarding from the analysis all proteins that were classified 'Mitochondria' or 'Nucleus' by TAGM analysis of the mito control cell line.

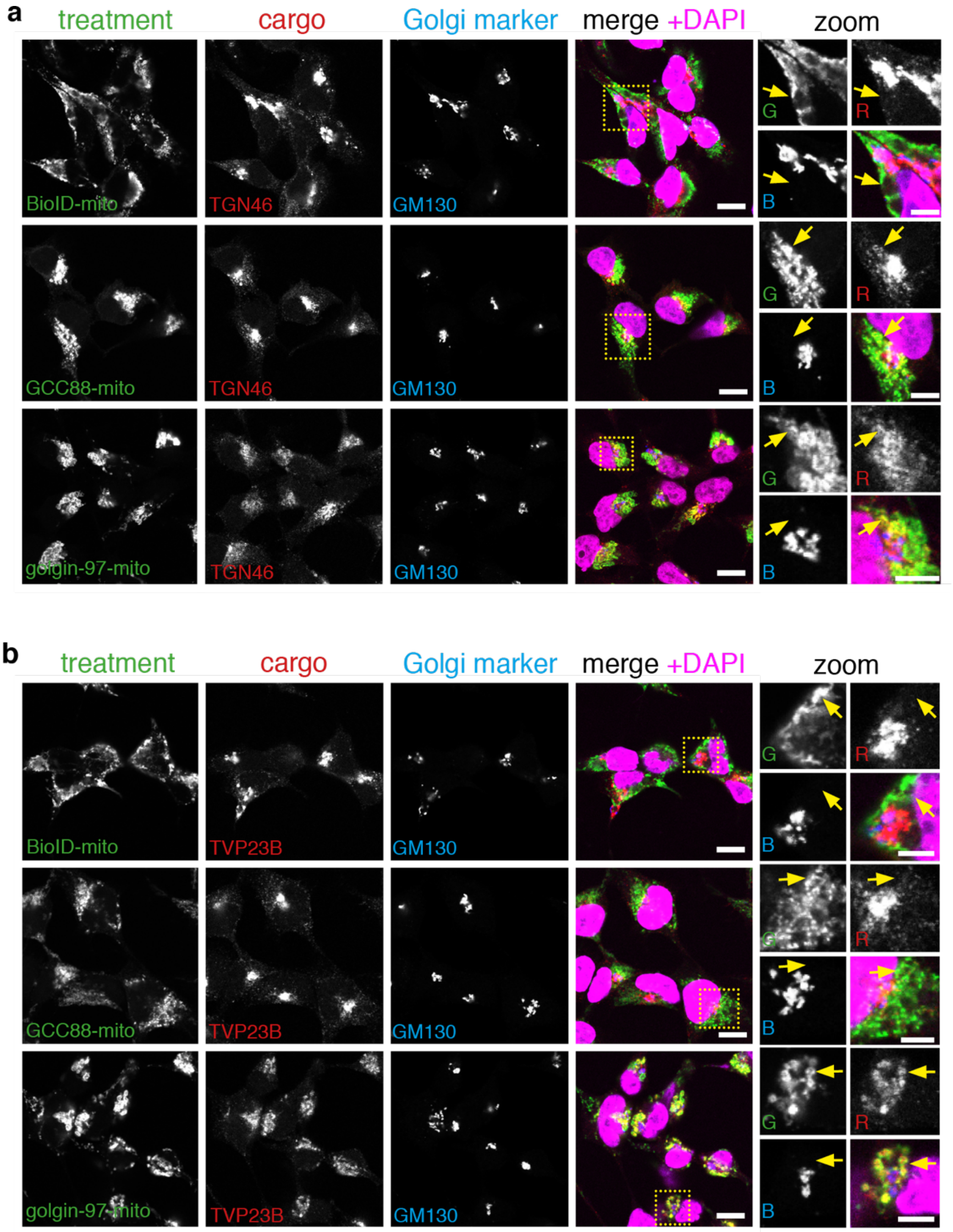

Supplementary Figure 2. Mito-relocation of cargo by golgin-97-mito and GCC88-mito.

a,b. Confocal micrographs of 293T cells stably expressing doxycycline-inducible BioID-mito, golgin-97-mito or GCC88-mito constructs and labelled with antibodies against the HA tag in the chimeras and endogenous TGN46 or TVP23B (cargo), and GM130 (marker of *cis*-Golgi). Expression was induced by 1 µg ml^{-1} doxycycline for 48 h, and micrographs are representative of three independent experiments. Scale bars, 10 µm (zooms 5 µm).

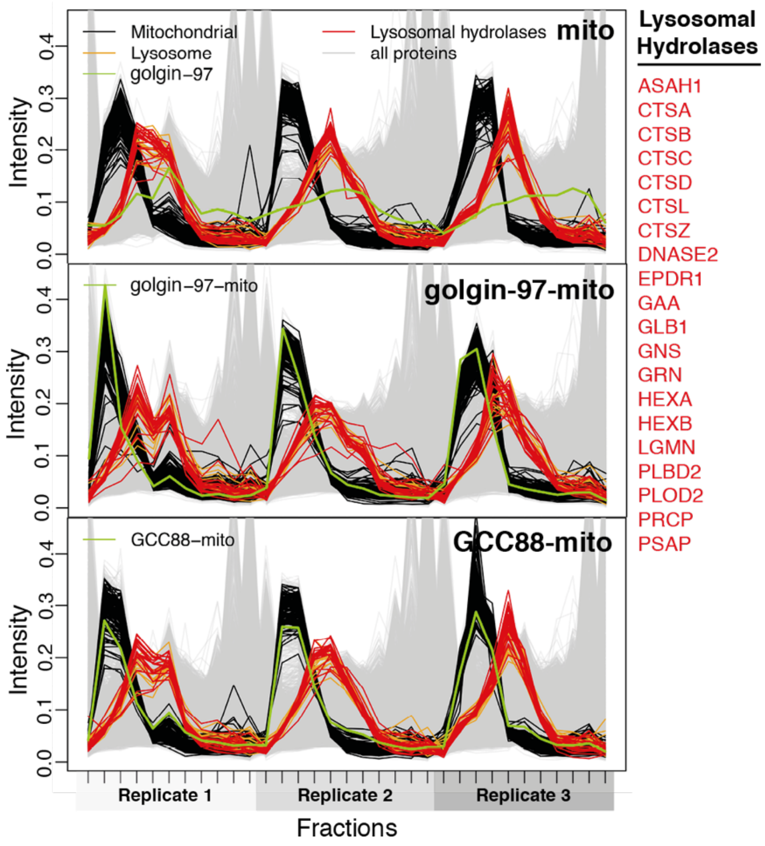

Supplementary Figure 3. Lysosomal hydrolases are not shifted to a mitochondrial profile by golgin-97-mito or GCC88-mito.

TMT reporter ion distributions of proteins across fractions for each replicate for mito, golgin-97-mito and GCC88-mito showing the profiles of known mitochondrial markers (Mitochondria), known lysosomal markers (Lysosome) and lysosomal hydrolases that have previously been shown to be found in anterograde Golgi-to-endosome clathrin/AP-1 vesicles[1, 2].

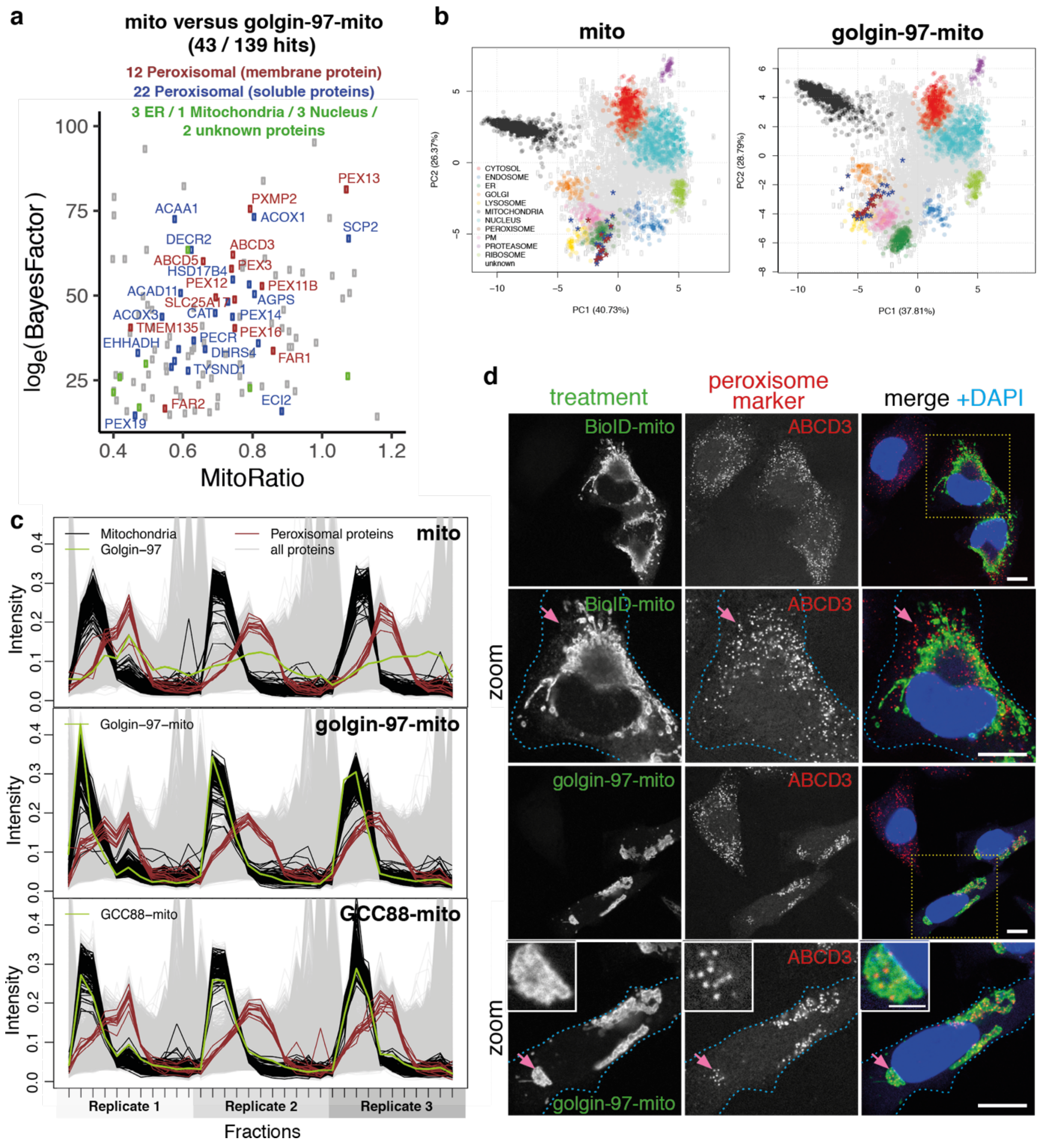

Supplementary Figure 4. Redirection of peroxisomes by mitochondrial golgins.
a. MitoRatio versus Bayes Factor analysis of mito versus golgin-97-mito showing all hits (brown, blue and green) that are not localised to the endosomal network. Classification of their localisation is indicated and is based on multiple localisation databases. **b.** PCA projections for the LOPIT-DC of mito versus golgin-97-mito showing SVM classifications along with peroxisomal membrane proteins (brown) and peroxisomal soluble proteins (blue) from (a). **c.** TMT reporter ion distributions of proteins across fractions for each replicate for mito, golgin-97-mito and GCC88-mito showing the profiles of known mitochondrial markers (Mitochondria) and peroxisomal membrane protein hits (a,b) (Peroxisomal Proteins). **d.** Representative confocal micrographs from three independent replicates of HeLa cells expressing BioID-mito and the indicated golgin-mito construct (HA-tag) stained for endogenous ABCD3 (peroxisome marker) and with DAPI. Scale bars, 10 µm (inset 2.5 µm).

Supplementary Table 1. Primers used in this study.

Name	Sequence
FurinC'_F	5'GAACTGATCAATGGAGCTGAGGCCCTGGTTGCTATGGGT GGTAGCAGCAACAGGAACCTT3'
FurinC'_R	5'CTATACCGGTGAGGGCGCTCTGGTCTTTGATAAAGGCGG TCCTCTCGCCCCGGCCCTCGT3'
TMEM87C'_F	5'CATTGGATCCGCCACCATGGCTGCTGCTGCTTGGTTACA AGTTTTACCTGTTATTTTATT3'
TMEM87C' _R	5'GAATACCGGTTTCCATTTTAGAACGTTCAAAATGAGTAATC ATACGTTCTTCATCAGAAT3'
G97_F	5'TATCGCTAGCGCGGCCGCACAGCAAACATGTTTGCAAAA CTGAAGAAGAAAATTGCAGAA3'
G97ΔC_R	5'CTGCGGATCCTGCGTAGTCGGGCACGTCGTAGGGGTACA TGTTTGCTGTGGTACCGACGG3'
GCC88_F	5'ACTTAAGCTTACCGGTCTCAAGATGGAGAAGTTTGGGATG AATTTCGGGGGCGGCCCGAG3'
GCC88ΔC_R	5'CTGTGGTACCGGCTCCCTCCCTGCTCTGGTCCCTGATGT TCTTTTCGATGTGGCTCTGCA3'
Mito_F	5'TATCGCTAGCGCTGGTACCACAGCAAACATGTACCCCTAC GACGTGCCCGACTACGCAGG3'
Mito_R	5'CGAGCCCGGGTCAAGACCGTGGCAGGAGCTTGTATTTGT ACAGCACAAACCCCAGGGCAG3'
BioIDC' _F	5'CAATGCTAGCGCCGCCACCATGTTCAAGAACCTGATCTG GCTGAAGGAGGTGGACAGCAC3'
BioIDC' _R	5'GAATGGTACCGCTTCTTCTCAGGCTGAACTCGCCGCTCA GGATCTCCTTGATGCCCTCCT3'
G97cas9Top	5'CACCGTGAACAGATACGGAAGTTAG3'
G97cas9Bottom	5'AAACCTAACTTCCGTATCTGTTCAC3'
G245cas9Top	5'CACCGGTCCTGCTCCTCATTCTTGT3'
G245cas9Bottom	5'AAACACAAGAATGAGGAGCAGGACC3'
GCC88cas9Top	5'CACCGTCAGCAGGCTTTTATAGGCA3'
GCC88cas9Bottom	5'AAACTGCCTATAAAAGCCTGCTGAC3'
TBC1D23cas9Top	5'CACCGCTGCCAACGTCGAGCGGCGA3'
TBC1D23cas9Bottom	5'AAACTCGCCGCTCGACGTTGGCAG3'

Supplementary Table 2. Primary antibodies used in this study

Antigen	Species	Dilution[1]	Source	Identifiers[2]
HA (3F10)	Rat monoclonal	IF 1:300<br>WB 1:1000	Roche	11 867 423 001<br>RRID:AB_390918
TGN46	Sheep polyclonal	IF 1:300<br>WB 1:2000	ABD serotec	AHP500G<br>RRID:AB_323104
ATG9A	Rabbit monoclonal	IF 1:200	Abcam	Ab108338<br>RRID:AB_10863880
TVP23B	Rabbit polyclonal	IF 1:200	Human Protein Atlas	HPA019585<br>RRID:AB_1848385
GM130	Mouse monoclonal	IF 1:300	BD Transduction Labs	610823<br>RRID:AB_398142
TMEM87A	Rabbit polyclonal	IF 1:200	Human Protein Atlas	HPA018104<br>RRID:AB_1858135
β-actin	Rabbit polyclonal	WB 1:3000	Abcam	Ab8227<br>RRID:AB_2305186
ABCD3	Mouse monoclonal	IF 1:300	Atlas Antibodies	AMAb90995<br>RRID:AB_2665755
GCC88	Rabbit polyclonal	IF 1:200<br>WB 1:2000	Human Protein Atlas	HPA021323<br>RRID:AB_1849554
golgin-97	Rabbit polyclonal	IF 1:200<br>WB 1:2000	Human Protein Atlas	HPA044329<br>RRID:AB_2678897
golgin-245	Mouse monoclonal	IF 1:200<br>WB 1:1000	BD Transduction Labs	611281<br>RRID:AB_398809

1: IF, immunofluorescence, WB, western blotting

2: Supplier catalog number and Research Resource Identifier (RRID) from the Resource Identification Portal.

Supplementary References

1. Hirst, J. *et al.* Contributions of epsinR and gadkin to clathrin-mediated intracellular trafficking. *Mol Biol Cell* **26**, 3085-3103 (2015).
2. Navarro Negredo, P. *et al.* Contribution of the clathrin adaptor AP-1 subunit micro1 to acidic cluster protein sorting. *J Cell Biol* **216**, 2927-2943 (2017).
