# Supplementary material for: Spatial proteomics defines the content of trafficking vesicles captured by golgin tethers
Source: Nat Commun. 2020 Nov 25;11:5987. doi: 10.1038/s41467-020-19840-4 (PMC7689464; doi:10.1038/s41467-020-19840-4)
Supplement: Supplementary file 3 — Description of Additional Supplementary Files [file 41467_2020_19840_MOESM3_ESM.pdf]

Description of Additional Supplementary Files

File Name: Supplementary Data 1
Description: LOPIT-DC Mass spectrometry data. This table provides the abundance of TMT-labelled peptides and overall properties of each quantified protein across three independent replicates for each LOPIT-DC treatment after analysis using ProteomeDiscoverer. TMT labelling of the LOPIT fractions is as indicated in Fig. 1b. .xlsx file.

File Name: Supplementary Data 2
Description: LOPIT-DC data analysis.

File Name: Supplementary Movie 1
Electron microscopic tomogram of a Lowicryl-embedded HeLa cell expressing golgin-97-mito and TMEM87A-RFP, throughout a thickness of approximately 350 nm. The 3D segmentation model is shown as an overlay. The mitochondrial surfaces are drawn in purple and the circular vesicles accumulated between them are drawn in green. .mov file.
