# Supplementary material for: Spatial proteomics defines the content of trafficking vesicles captured by golgin tethers
Source: Nat Commun. 2020 Nov 25;11:5987. doi: 10.1038/s41467-020-19840-4 (PMC7689464; doi:10.1038/s41467-020-19840-4)
Supplement: Supplementary file 7 — Reporting Summary [file 41467_2020_19840_MOESM7_ESM.pdf]

nature research

Corresponding author(s): John Shin and Sean Munro

Last updated by author(s): Sep 25, 2020

Reporting Summary

Nature Research wishes to improve the reproducibility of the work that we publish. This form provides structure for consistency and transparency in reporting. For further information on Nature Research policies, see Authors & Referees and the Editorial Policy Checklist .

Statistics

For all statistical analyses, confirm that the following items are present in the figure legend, table legend, main text, or Methods section.

n/a	Confirmed	
☐	☒	The exact sample size (*n*) for each experimental group/condition, given as a discrete number and unit of measurement
☐	☒	A statement on whether measurements were taken from distinct samples or whether the same sample was measured repeatedly
☐	☒	The statistical test(s) used AND whether they are one- or two-sided<br>*Only common tests should be described solely by name; describe more complex techniques in the Methods section.*
☐	☒	A description of all covariates tested
☐	☒	A description of any assumptions or corrections, such as tests of normality and adjustment for multiple comparisons
☐	☒	A full description of the statistical parameters including central tendency (e.g. means) or other basic estimates (e.g. regression coefficient) AND variation (e.g. standard deviation) or associated estimates of uncertainty (e.g. confidence intervals)
☐	☒	For null hypothesis testing, the test statistic (e.g. *F*, *t*, *r*) with confidence intervals, effect sizes, degrees of freedom and *P* value noted<br>*Give P values as exact values whenever suitable.*
☐	☒	For Bayesian analysis, information on the choice of priors and Markov chain Monte Carlo settings
☒	☐	For hierarchical and complex designs, identification of the appropriate level for tests and full reporting of outcomes
☒	☐	Estimates of effect sizes (e.g. Cohen's *d*, Pearson's *r*), indicating how they were calculated

Our web collection on statistics for biologists contains articles on many of the points above.

Software and code

Policy information about availability of computer code

Data collection	LOPIT-DC mass-spectrometry data was collected with Proteome Discoverer v2.1 (Thermo Scientific).
Data analysis	Analysis of LOPIT-DC mass-spectrometry was Bioconductor 3.10 and R 3.6. EM data was analysed with Amira v4.10.29 (Thermo Scientific), vIMOD 4.10.29 and vSerialEM 3.8.0. Vesicle sizes were analysed with the Image J LimeSeg plug-in v0.4.4. Full details are provided in the Methods section.

For manuscripts utilizing custom algorithms or software that are central to the research but not yet described in published literature, software must be made available to editors/reviewers. We strongly encourage code deposition in a community repository (e.g. GitHub). See the Nature Research guidelines for submitting code & software for further information.

Data

Policy information about availability of data

All manuscripts must include a data availability statement. This statement should provide the following information, where applicable:

- Accession codes, unique identifiers, or web links for publicly available datasets
- A list of figures that have associated raw data
- A description of any restrictions on data availability

Mass spectrometry data used in this study are summarised in Supplementary Data files 1 and 2. All protein-level datasets generated during this study are available in the Bioconductor pRolocdata package (version ≥ 1.25.2) at https://github.com/lgatto/pRolocdata. Interactive versions of the PCA plots can be viewed online through dedicated R Shiny apps at https://proteome.shinyapps.io/golgins_mito/, https://proteome.shinyapps.io/golgins_golgin97mito/ and https://proteome.shinyapps.io/golgins_gcc88mito/. The mass spectrometry proteomics data have been deposited to the ProteomeXchange Consortium via the PRIDE75 partner repository with the dataset identifier PXD018110 [https://www.ebi.ac.uk/pride/archive/projects/PXD018110]. The source data underlying Figs 1c, 1g, 2f, 4b-d, 6a, 6c and 6d are provided as a Source Data file. Protein localisation data from UniProt [https://www.uniprot.org/help/subcellular_location] and the Human Protein Atlas [https://www.proteinatlas.org/] were obtained using the BioMart portal [https://www.ensembl.org/info/data/biomart/index.html]68. All reagents generated by this study are available from the corresponding authors on request.

Field-specific reporting

Please select the one below that is the best fit for your research. If you are not sure, read the appropriate sections before making your selection.

[x] Life sciences   [ ] Behavioural & social sciences   [ ] Ecological, evolutionary & environmental sciences

For a reference copy of the document with all sections, see nature.com/documents/nr-reporting-summary-flat.pdf

Life sciences study design

All studies must disclose on these points even when the disclosure is negative.

Sample size	N was chosen to be sufficient for statistical significance over a wide range of possible effect sizes based on experience and similar published studies. Sample sizes are indicated for each experiment in the Figure Legends, and all data are provided in the Source Data file.
Data exclusions	As described in the Methods, during the LOPIT-DC analysis of proteins that were relocalised by the the mitochondrial golgins, those proteins predicted to be mitochondria or nuclear were excluded from the analysis as they are not relevant to vesicular cargo. These excluded proteins are stated in Supplementary Data 2.
Replication	All experimental findings were reliably reproduced at least twice, and the number of such replicates is stated in the Figure Legends.
Randomization	Randomization was not applicable because we were comparing two cell lines where there was only a single difference between each cell population - ie the presense or absence of a mitochondrial golgin. There were thus no possible confounding factors.
Blinding	Most experiments used objective quantitative assays and so blinding was not required. For immunofluorescence experiments where cells were selected for imaging, blinding was not felt necessary based upon experience and similar published studies. All experiments were repeated at least twice to ensure reproducibility, and typically examined by multiple authors. The number of replicates is stated in the Figure Legends.

Reporting for specific materials, systems and methods

We require information from authors about some types of materials, experimental systems and methods used in many studies. Here, indicate whether each material, system or method listed is relevant to your study. If you are not sure if a list item applies to your research, read the appropriate section before selecting a response.

Materials & experimental systems

n/a	Involved in the study	
[ ]	[x]	Antibodies
[ ]	[x]	Eukaryotic cell lines
[x]	[ ]	Palaeontology
[x]	[ ]	Animals and other organisms
[x]	[ ]	Human research participants
[x]	[ ]	Clinical data

Methods

n/a	Involved in the study	
[x]	[ ]	ChIP-seq
[x]	[ ]	Flow cytometry
[x]	[ ]	MRI-based neuroimaging

Antibodies

Antibodies used	As stated in Supplementary Table 2:<br>HA (3F10) Rat monoclonal IF 1:300 WB 1:1000 Roche 11 867 423 001 RRID:AB_390918<br>TGN46 Sheep polyclonal IF 1:300 WB 1:2000 ABD serotec AHP500G RRID:AB_323104<br>ATG9A Rabbit monoclonal IF 1:200 Abcam Ab108338 RRID:AB_10863880<br>TVP23B Rabbit polyclonal IF 1:200 Human Protein Atlas HPA019585 RRID:AB_1848385<br>GM130 Mouse monoclonal IF 1:300 BD Transduction Labs 610823 RRID:AB_398142<br>TMEM87A Rabbit polyclonal IF 1:200 Human Protein Atlas HPA018104 RRID:AB_1858135<br>β-actin Rabbit polyclonal WB 1:3000 Abcam Ab8227 RRID:AB_2305186<br>ABCD3 Mouse monoclonal IF 1:300 Atlas Antibodies AMAb90995 RRID:AB_2665755<br>GCC88 Rabbit polyclonal IF 1:200 WB 1:2000 Human Protein Atlas HPA021323 RRID:AB_1849554<br>golgin-97 Rabbit polyclonal IF 1:200 WB 1:2000 Human Protein Atlas HPA044329 RRID:AB_2678897<br>golgin-245 Mouse monoclonal IF 1:200 WB 1:1000 BD Transduction Labs 611281 RRID:AB_39880
Validation	The above list provides for each antibody the Research Resource Identifier (RRID) from the Resource Identification Portal which provides information about validation and previous publications.

Eukaryotic cell lines

Policy information about cell lines

Cell line source(s)	HeLa cells were from the American Type Culture Collection, Flp-In 293 T-REx cells were from ThermoFisher Scientific.
Authentication	Cell lines authentification was not performed for the study.
Mycoplasma contamination	All lines tested negative for mycoplasma, using MycoAlert from Lonza
Commonly misidentified lines (See ICLAC register)	No commonly misidentified lines were used.
